# Small Bowel Obstruction Secondary to an Ingested Iron “Stay Fresh” Pack

**DOI:** 10.7759/cureus.90419

**Published:** 2025-08-18

**Authors:** Robinson D Taylor, Zachary Kehrt

**Affiliations:** 1 Emergency Medicine, Sutter Roseville Medical Center, Roseville, USA

**Keywords:** bowel obstruction, emergency medicine, ingested foreign body, iron, silica, stay fresh packet

## Abstract

"Stay-fresh" packets are commonly used items to prevent oxygen or water from damaging items. These are routinely placed in food items such as meat jerky, nuts, dried fruits, and breads, and are usually not toxic when ingested. While a few case reports have described esophageal obstruction following the ingestion of silica stay-fresh packets, no case reports to date have documented pathology resulting from the ingestion of iron stay-fresh packets in otherwise healthy individuals.

In this case report, a 51-year-old man presented to the ER with abdominal pain. The CT scan indicated severe proximal bowel distention with distal enteritis, along with a possible small bowel foreign body. The patient denied ingesting any foreign bodies. Abdominal exam gradually worsened, and an exploratory laparotomy was performed, revealing an obstruction secondary to an ingested iron “stay-fresh” packet.

## Introduction

Consumer items, including shelf-stable food, clothing, and furniture, are frequently designed to be stored for periods of time while withstanding degradation from oxygen and moisture. Oxygen within the air can cause spoilage of food through a multitude of ways, including oxidation of ingredients to off-putting flavors, loss of flavor, and microbial growth (bacterial and fungal). Many methods of food preservation involve packaging food in a low-oxygen environment that can be sealed or maintained through goods’ shelf life. One such storage technique is the inclusion of iron or silica in small containers or “packets” within the packaging to absorb oxygen or water, respectively [1]. Iron “stay fresh” or “freshness” packets utilize ferrous oxide with Iron (II) or Fe2+. When ferrous oxide comes in contact with oxygen, it will oxidize to ferric oxide (FeO → Fe2O3), removing a proportional amount of oxygen from the air, and maintaining a low oxygen environment within a sealed container. Generally, 2.2g of ferric oxide produced will remove approximately 100cc of oxygen from a sealed environment [1].

The inclusion of ferrous oxide in small, sealed packets within food packaging has long been considered a consumer-safe method for preserving food without significant risk to the end user. Multiple safeguards exist to prevent accidental ingestion of iron packets when included with food: the packets are generally distinct from the food they are contained with, the packets are clearly labeled “do not eat,” and the material of the packets is often hard to chew and inherently difficult to swallow. For reference, the LD50 of ferrous iron is 16 g/kg body weight or 1,120g for a 70 kg person [2]. A standard-size iron oxygen absorber packet contains 2.5 g of iron. A 70 kg person would need to ingest 448 packets to reach the 1,120 g LD50. If the ferric oxide in a single packet were swallowed independently of the packaging, the general recommendation is for the patient to hydrate thoroughly without any further follow-up needed. In the event a packet is swallowed intact or chewed, imaging is advised with follow-up endoscopy to remove the packet [1].

Bowel obstructions are a relatively common presentation with an incidence of 350,000 cases/year in the US [3]. The leading cause of bowel obstructions in the US is adhesions [4]. Approximately 20% of bowel obstructions require surgery. Approximately 2-4% of all emergency room visits are due to obstruction, and they are the reason for 15% of hospital admissions. They account for roughly $3 billion worth of patient care in 2005 [5], a cost that likely has risen through the years.

There have been only a few case reports of patients ingesting stay-fresh packets, and the majority of these describe obstructions that are pre-pyloric and involve bowel with strictures present. These reports are sparse and span decades, and also involve silica desiccant packets. In 1980, Muhletaler et al. describe two patients who developed a partial obstruction in the pylorus due to an underlying stricture after ingesting a pill bottle desiccant [6]. It is unclear if these were silica or a different type of material. Additionally, the desiccant recovered was cylindrical in shape. Wu et al. similarly discuss a pharmaceutical desiccant causing a small bowel obstruction in a patient with underlying Crohn’s disease in 1998 [7]. Again, the paper did not identify the composition of the desiccant, and the patient had pre-existing strictures. In 2015, a case report by Barkin and Barkin describes a pill bottle desiccant causing an obstruction in a patient with an underlying esophageal stricture [8]. Lassiter et al. in 2020 also report an esophageal obstruction in a patient who ingested a silica pill bottle desiccant [9]. However, this is among the first case reports of these packets causing a small bowel obstruction that is post-pyloric with otherwise normal underlying anatomy, or involving an iron stay fresh packet.

## Case presentation

A 51-year-old man with no significant past medical history presented to Sutter Roseville Medical Center Emergency Room with abdominal pain for five days. His pain initially started with sudden exertion. His last bowel movement was three days prior and was described as “liquidy.” The pain was characterized as a “gaseous feeling.” He also reported associated nausea but without vomiting. He endorsed a history of recurrent constipation requiring laxative use in the past. Otherwise, he denied any past medical history. He denied any history of abdominal surgeries. He admitted to drinking 12 beers per week. The exam was significant for diffuse abdominal tenderness, but he was nonperitonitic on palpation.

**Figure 1:**
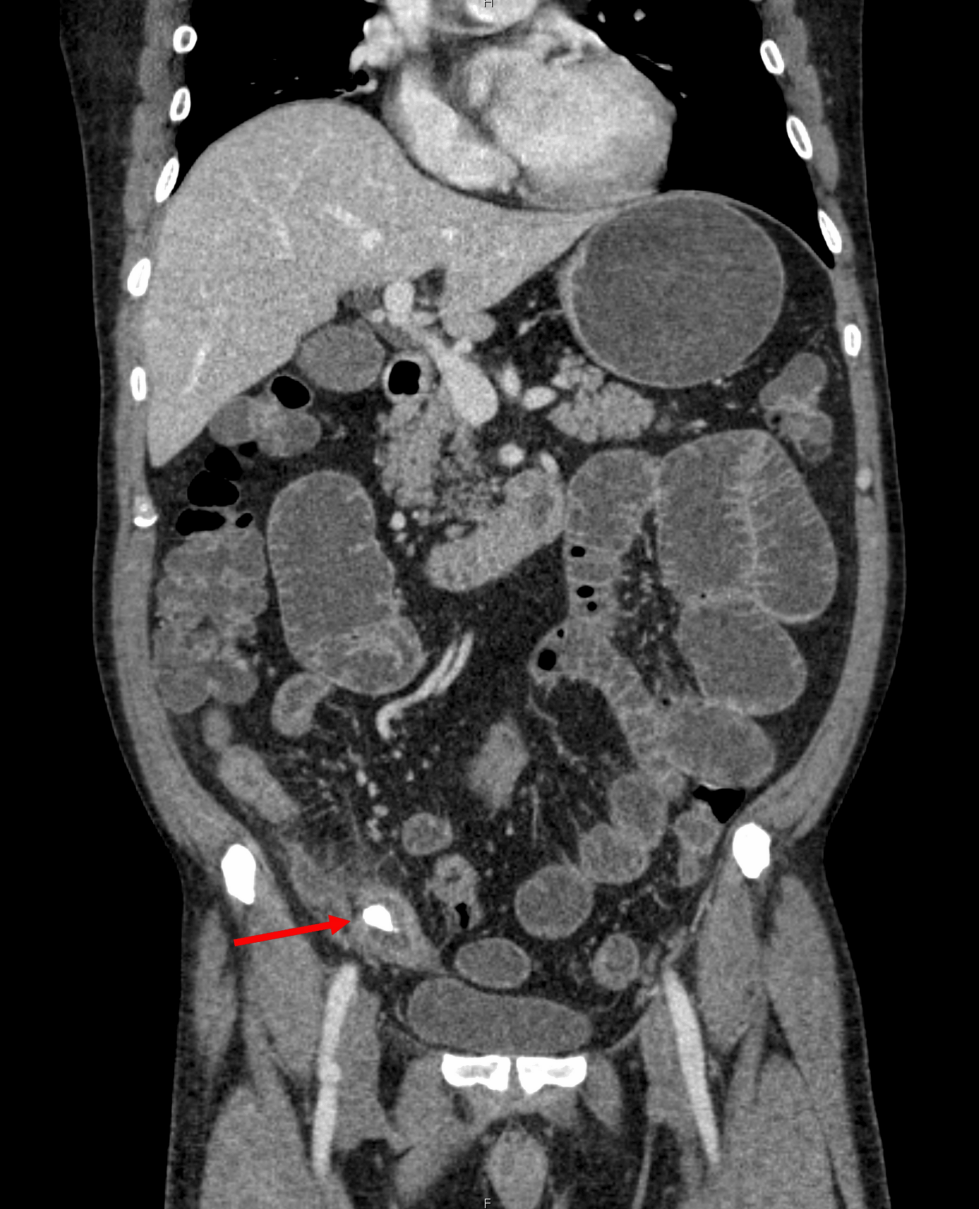
Patient’s computed tomography revealing a foreign body (red arrow) in the ileum with proximal small bowel dilation.

**Figure 2:**
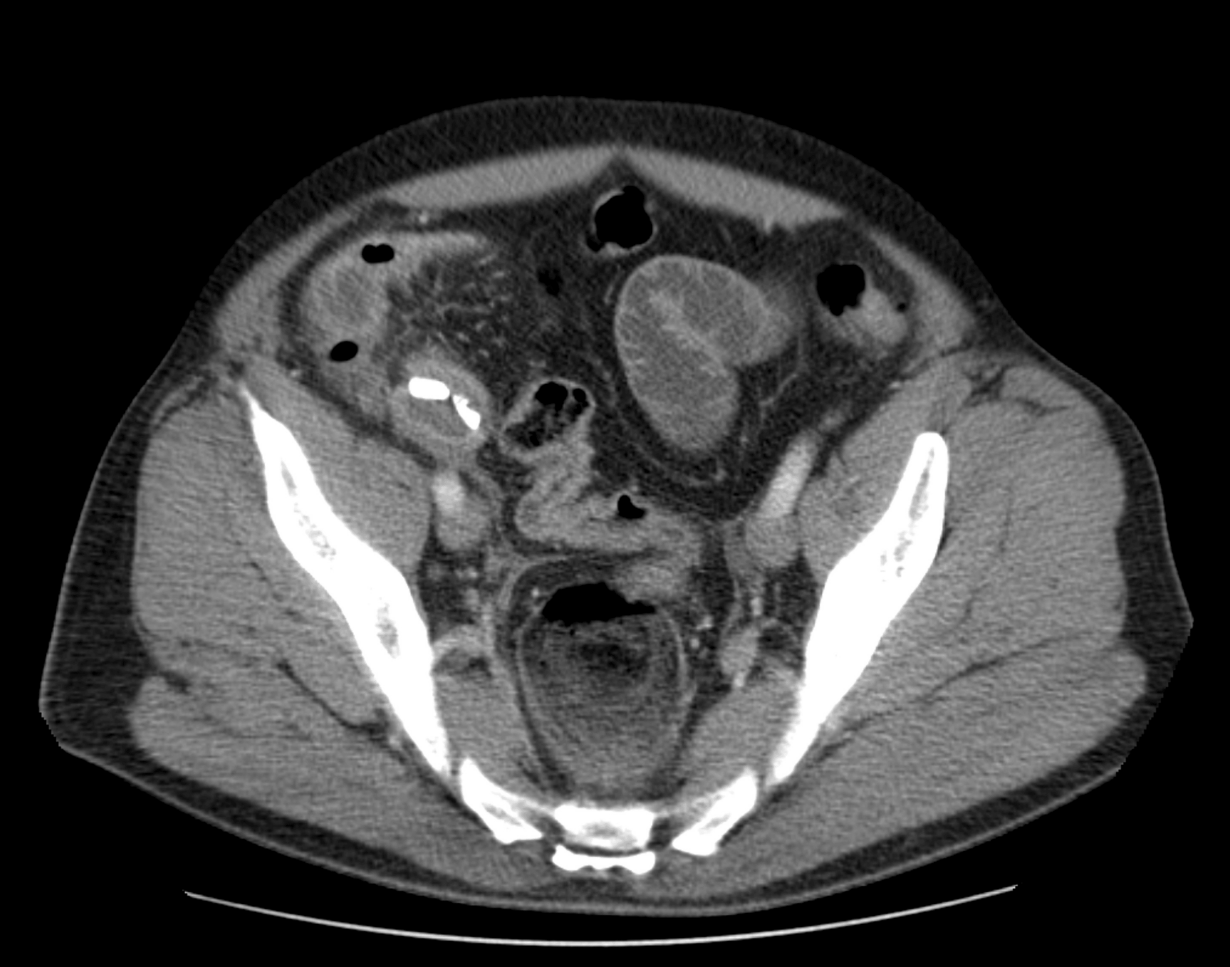
Transverse view of CT scan with the foreign body pictured

His workup included complete blood count (CBC), complete metabolic panel (CMP), lipase, and prothrombin time (PT). CMP was notable for hyponatremia. CBC was notable for a leukocytosis, with a low absolute neutrophil count (ANC), but a significant bandemia (Table 1). A CT scan was obtained, which revealed marked proximal small bowel distention with changes of distal small bowel enteritis, with the distal small bowel less distended but showing possible wall enhancement and mild thickening (Figures 1, 2). There was also a noted calcific-appearing density within the distal small bowel loops, without definite adjacent upstream small bowel distention to suggest an obstructive lesion. This was thought to be a possible swallowed foreign body versus an ingested medication.

**Table 1:** Laboratory results and normal values

Test		Result	Normal Range
Complete blood count	White blood cells	12.3 K/uL	4.0-11 K/uL
	Hemoglobin	15.4 g/dL	13.5-18 g/dL
	Hematocrit	43.2%	40-52%
	Platelets	313 k/uL	150-400 K/uL
	Absolute neutrophil count	0.9 K/uL	2.0 – 8.0 K/uL
	Absolute neutrophil bands	7.6 K/uL	0.0 – 1.4 K/uL
CMP	Sodium	128 mmol/L	136-145 mmol/L
	Potassium	3.7 mmol/L	3.5-5.1 mmol/L
	Chloride	91 mmol/L	98-107 mmol/L
	Bicarbonate	29 mmol/L	21-32 mmol/L
	Glucose	133 mg/dL	70-100 mg/dL
	Blood urea nitrogen	48 mg/dL	6-25 mg/dL
	Creatinine	1.21 mg/dL	0.50-1.30 mg/dL
	Estimated glomerular filtration rate	73	>60
	Calcium	8.8 mg/dL	8.2-10.2 mg/dL
	Total protein	7.9 g/dL	6.4-8.2 g/dL
	Albumin	3.2 g/dL	3.2-4.7 g/dL
	Total bilirubin	0.8 g/dL	<1.1 g/dL
	Alkaline phosphatase	88 U/L	26-137 U/L
	Aspartate aminotransferase	31 U/L	0-31 U/L
	Alanine aminotransferase	148 U/L	12-78 U/L
Lipase		12 U/L	13-77 U/L
Prothrombin time		14.8 seconds	11.5-15.1 seconds
International normalized ratio		1.2	0.9-1.2

Hematology was consulted, given the low ANC and bandemia, and suggested this could be early leukemia versus an early septic response. Their recommendation was to obtain a repeat CBC in 24 hours to assess any changes in the white blood cell count.

On reassessment, the patient appeared to be stable. His pain had improved with morphine and fluids. He was questioned regarding the possible foreign body and adamantly denied ingesting anything other than food. When a PO challenge was attempted, he was unable to tolerate food. Given the low ANC, leukocytosis, inability to tolerate PO, and overall unclear clinical picture, the decision was made to admit the patient to the observation unit for repeat labs and serial abdominal exams. Surgery was not initially consulted as the patient had denied ingesting a foreign body, and his abdominal pain had improved. The next day, his exam worsened, and the patient’s abdomen became more distended during rounds. Acute care surgery was consulted, and given their concern for a small bowel obstruction, recommended NG tube placement and admission to medicine. After reviewing the CT scan, Surgery believed the finding of a foreign body may have been previously ingested medications, but it did not appear to be at the transition point of the small bowel obstruction and may be an incidental finding. Later that day, the patient consented to an exploratory laparotomy as they continued not to pass flatus.

It was found that the patient had an intraluminal foreign body about 20 centimeters (cm) from the ileocecal valve. 15 cm proximal to that, there appeared to be an area of small bowel with a circular patch of fibrinous exudate that appeared to be a sealed perforation. The foreign body was removed and was described as a "stay-fresh pack." The bowel was resected, and anastomosis was performed with an 80 mm purple load gastrointestinal anastomosis stapler. The resected bowel and the foreign object were sent to Pathology. The foreign body was determined to be a 3.5 x 2.5 x 0.3 cm white plastic pouch containing granular material displaying the following inscriptions: "PACKET ENCLOSED TO KEEP PRODUCT FRESH. DO NOT EAT PACKET. CONTAINS IRON." No accompanying pathology image was provided. The bowel submitted for path report was 5 cm x 2.5 cm. There was a 1 cm perforation site. The submitted bowel contained inflammation and ischemic changes. 

The patient was taken to PACU and eventually back to his med-surg floor. His initial leukocytosis peaked at 19.3 before downtrending. His initial low ANC resolved and appropriately uptrended. The patient recovered well and was discharged home.

## Discussion

In this case, we describe a patient who developed a small bowel obstruction from an intact iron oxygen scavenger packet. There was no mention in the pathology report that the packet itself had been damaged or was otherwise not intact.

From a toxicity perspective, the contents of the packet were far below the LD50 for Ferrous oxide. Iron toxicity has been well described and follows a predictable course [2]. Around 30 minutes to 6 hours after ingestion, patients will typically complain of abdominal pain, nausea, and vomiting. For the next 24 hours, patients will usually remain asymptomatic, and this is followed by an anion gap acidosis, shock, and hepatotoxicity [2]. Iron poisoning has been associated with bowel obstructions, but this will usually occur two to eight weeks after ingestion. The most common area for obstruction is the gastric outlet, as this is where the iron tablets tend to collect and cause local scarring [2].

A patient’s risk of small bowel obstruction is increased with age, history of obstruction, and previous surgeries. The most common cause of obstructions is adhesions, which usually occur secondary to previous intrabdominal surgeries. Other common causes include inflammatory bowel disease, hernias, tumors, and infectious diseases. In a patient without these risk factors, it is exceptionally rare to suffer from an obstruction. To date, no case reports have described an oxygen absorber packet causing a distal intestinal obstruction in the setting of normal bowel anatomy [3-5]. Further, in patients with normal bowel anatomy, previous case reports have only found these obstructions to occur proximal to the pylorus [6-9].

## Conclusions

Iron oxygen absorber packets are commonly found in many food items to prevent spoilage. While these are generally appreciated as being safe for consumers, it appears that they pose a risk of causing a small bowel obstruction if ingested. Given the small amount of iron contained in these packets, a patient would need to ingest an exorbitant amount of them to display any sort of iron toxicity. To date, this is the first case report of an ingestion of these packets causing a small bowel obstruction in an otherwise healthy patient. Previous literature has described an obstruction from stay-fresh packets proximal to the gastric outlet, or obstructions in patients with underlying bowel disease or pre-existing strictures. In these case reports, the packets involved contained silica, not iron. While iron poisoning can cause obstructions, this is due to scarring of the bowel from iron irritation of the mucosa. Finally, this case highlights that a foreign body causing an obstruction should be considered in an unexplained obstruction, even if there is no ingestion history. 
